# Cooperative Positioning Method of a Multi-UAV Based on an Adaptive Fault-Tolerant Federated Filter

**DOI:** 10.3390/s23218823

**Published:** 2023-10-30

**Authors:** Pengfei Zhang, Zhenhua Ma, Yin He, Yawen Li, Wenzheng Cheng

**Affiliations:** 1 School of Aerospace Engineering, North University of China, Taiyuan 030051, China; 2 Intelligent Weapon Research Institute, North University of China, Taiyuan 030051, China

**Keywords:** multi-UAV formation, cooperative positioning, adaptive fault-tolerant federated filter, fault-tolerant performance

## Abstract

Aiming at the problem of the low cooperative positioning accuracy and robustness of multi-UAV formation, a cooperative positioning method of a multi-UAV based on an adaptive fault-tolerant federated filter is proposed. Combined with the position of the follower UAV and leader UAV, and the relative range between them, a cooperative positioning model of the follower UAV is established. On this basis, an adaptive fault-tolerant federated filter is designed. Fault detection and isolation technology are added to improve the positioning accuracy of the follower UAV and the fault tolerance performance of the filter. Meanwhile, the measurement noise matrix is adjusted by the adaptive information allocation coefficient to reduce the impact of undetected fault information on the sub-filter and global estimation accuracy. The simulation results show that the adaptive fault-tolerant federated algorithm can greatly improve the positioning accuracy, which is 83.4% higher than that of the absolute positioning accuracy of a single UAV. In the case of a gradual fault, the method has a stronger fault-tolerant performance and reconstruction performance.

## 1. Introduction

UAVs have the characteristics of a low cost, simple operation and easy deployment, which not only saves a lot of manpower and material resources but also makes them less restricted by the environment and plays a great role in military and civilian fields [1,2]. However, in recent years, the tasks that need to be performed by UAVs have become more and more complex, and the environment has become more and more extensive. A single UAV is prone to mission failure due to its small coverage area and failure of the UAV. Inspired by natural biological clusters, UAVs have gradually developed into cluster formations and have great potential application value in battlefield reconnaissance, disaster relief, communication relay, 3D reconstruction and other fields [3,4,5,6,7]. Compared with a single UAV, a UAV swarm can fully take advantage of clusters, so that members can perform tasks in parallel. Through cooperation among members, UAV swarms broaden the way and field of task execution. They have the advantages of functional distribution, a high system survival rate and a high efficiency and have great potential application value [8,9,10].

In the process of multi-UAV formation flights, navigation and positioning are required for the whole flight process, and high-precision positioning is the key technology to ensure UAVs’ safety and path planning [11,12]. The existing mainstream navigation methods mainly rely on the combination of a high-precision inertial navigation system (INS) and a global navigation satellite system (GNSS) to achieve high precision [13]. However, due to cost and load constraints, each UAV cannot be equipped with high-precision navigation equipment. Therefore, in the case of load permitting, other low-cost sensors can be equipped for UAVs to perform cooperative positioning through information interaction among UAVs to improve the positioning accuracy of multi-UAV formation flights [14,15].

Multi-UAV cooperative positioning has become a hot topic for scholars at home and abroad. Chen M [16] proposed a hybrid cooperative navigation (CN) method for UAV swarm based on a factor graph and the Kalman filter. The global factor graph is used to combine the global navigation satellite system (GNSS) and ranging information to provide position estimation for the modified distributed Kalman filter; then, a distributed Kalman filter is established on each UAV to fuse inertial information and optimize position estimation and modify navigation states. Simulation results show that it can provide a more precise and efficient CN solution than traditional CN methods. Aiming at the real-time positioning requirements of UAV clusters, Tang C [17] proposed a multi-source fusion UAV cluster collaborative positioning method based on information geometry, which can effectively suppress abrupt errors and realize rapid positioning. Zhu X [18] proposed a cooperative positioning method following the motion vector of the UAV. The leader UAV obtains high-precision positioning information through the INS/GPS, and the follower UAV fuses the position information of the leader UAV and its own INS positioning information through the improved extended Kalman filter to improve its positioning accuracy. Wan J [19] proposed a dynamic nonparametric belief propagation (dNBP) algorithm to calculate the posterior distribution of the UAV’s position conditioned on all observations made in the entire UAV group. This method is suitable for dealing with nonlinear models and highly non-Gaussian distributions that appear in applications. However, the authors of the above articles have carried out a lot of research on the positioning accuracy of cooperative positioning but have not considered the fault detection and fault-tolerance performance of multi-UAV formation, and the algorithm implementation is very cumbersome.

For feedback control and sensor faults, Xu H [20] proposed an event-triggered predefined time decentralized output feedback control method, whereby they designed a predefined time filter to solve the computational complexity problem. Yu D [21] proposed a new adaptive fuzzy tracking control algorithm. This fault-tolerant control algorithm utilizes Lyapunov functions to ensure that all signals in the system are bounded when multiple faults occur. This paper considers feedback control and sensor failures and proposes a cooperative positioning method of a multi-UAV based on the adaptive fault-tolerant federated filter algorithm. The designed adaptive fault-tolerant federated filter is applied to fuse the positioning information of the follower UAV itself, the position information of the leader UAV and the relative ranging information of them for cooperative positioning. The information allocation coefficient is continuously adjusted according to the observation forecast residual, and the adaptive adjustment of the measurement noise matrix is realized by using the information allocation coefficient. Combined with fault detection and isolation technology, the global optimal estimation of positioning errors is realized. As a result, the positioning accuracy of the follower UAV is improved, and the fault tolerance performance of the system can also be guaranteed. The innovations and contributions of this paper are as follows:

(1) Aiming at the problem of the cumbersome algorithm of multi-UAV cooperative positioning, a federated filter is designed to realize cooperative localization in this paper. This method is simple and easy to implement and can improve the positioning accuracy of the follower UAVs.

(2) Based on the traditional federated filter, this paper introduces an adaptive information allocation coefficient to adjust the measurement matrix so as to change the utilization rate of observation data and improve the fault-tolerance performance of multi-UAV cooperative positioning.

## 2. The Principle of the Federated Filter and Cooperative Positioning Scheme

### 2.1. The Principle of the Federated Filter

**Figure 1:**
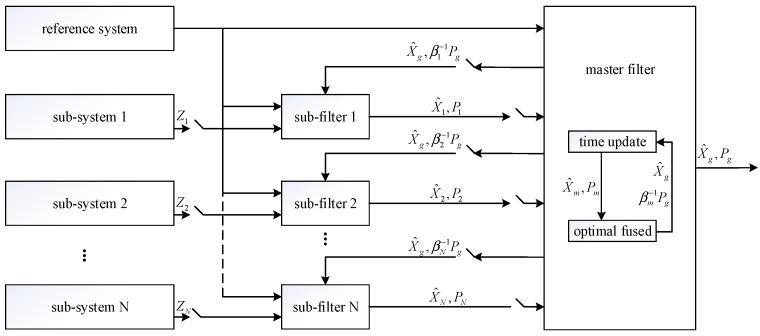
The general structure of the federated filter.

The federated filter is evolved on the basis of decentralized filtering, which is a two-stage filter. The general structure of the federated filter is shown in Figure 1 [22].

The federated filter is composed of multiple sub-filters and a master filter. The sub-filters process the data in parallel. The state estimates of them are acquired by their measurements, respectively; the state estimates ${\hat{X}}_{i}$ and the estimated covariance matrix $P_{i}$ are transmitted to the master filter as the input information. The master filter outputs the optimal solution of the state estimates $X_{g}$ and the covariance matrix $P_{g}$ after the optimal fusion of the sub-filter data. Meanwhile, the state estimates and covariance matrix of each sub-filter are reset by the obtained optimal result; $\beta_{i}(0\leq \beta \leq 1)$ is the information allocation coefficient [23]. The federated filter mainly fuses the estimated values and variances transmitted by the sub-filters, which is essentially a weighted average.

### 2.2. Multi-UAV Cooperative Positioning Scheme

**Figure 2:**
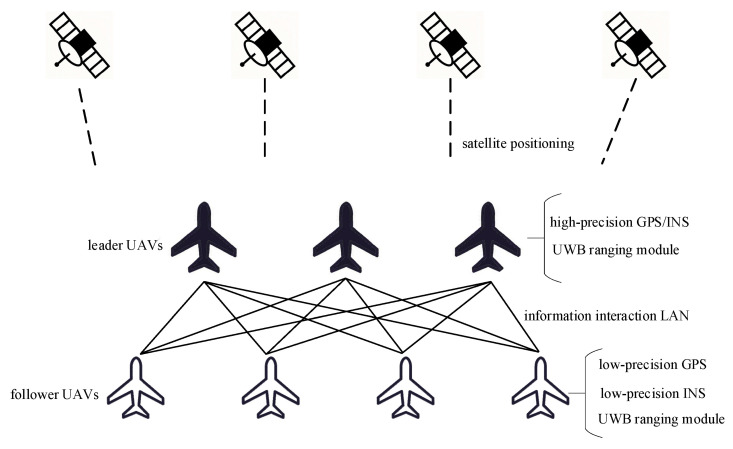
Multi-UAV cooperative positioning scheme.

Aiming at the cooperative positioning problem of a multi-UAV, this paper designs a cooperative positioning scheme of follower UAVs following leader UAVs. In this scheme, a small number of UAVs are equipped with high-precision GPS/INS integrated navigation equipment, and these UAVs are defined as the leader UAVs. The remaining UAVs are defined as the follower UAVs, which are equipped with low-precision GPS and INS navigation equipment. In addition, all follower UAVs and leader UAVs are equipped with ranging equipment. Based on the advantages of the UWB ranging module, such as strong anti-interference ability, fast data transmission speed and accurate ranging [24], this paper selects the UWB ranging module as the ranging equipment. The cooperative positioning scheme is shown in Figure 2.

The leader UAVs realize high-precision positioning through the high-precision GPS/INS carried on them and provide the reference points for the relative navigation of the follower UAVs. The follower UAVs use their own low-precision INS as a public navigation system. On the one hand, the INS and the low-precision GPS carried by the follower UAV itself realize integrated navigation. On the other hand, the INS fuses the position and relative range of the leader UAV to realize relative navigation and realizes the optimal fusion of the two through an adaptive fault-tolerant federated filter, thereby improving the positioning accuracy and fault-tolerant performance of the follower UAV. In this scheme, every follower UAV only performs range measurement with the nearest leader UAV for cooperative positioning, so the adaptive fault-tolerant federated filter generally has only two sub-filters, namely, sub-filter 1 and sub-filter 2.

## 3. Model of the Adaptive Fault-Tolerant Federated Filter

### 3.1. State Equation of the Adaptive Fault-Tolerant Federated Filter

The INS was adopted for the public system of the adaptive fault-tolerant federated filter, so it was decided to select the output parameter error of the INS and the inertial instrument error as the state quantity of the two sub-filters. The east–north–up (ENU) geographic coordinate system was selected as the navigation coordinate system, and the state quantity is:

(1) $$X={\left[\varphi_{E},\varphi_{N},\varphi_{U},\delta v_{E},\delta v_{N},\delta v_{U},\delta L,\delta \lambda ,\delta h,\varepsilon_{bx},\varepsilon_{by},\varepsilon_{bz},\nabla_{bx},\nabla_{by},\nabla_{bz}\right]}^{T}$$

where $\varphi_{E},\varphi_{N},\varphi_{U}$ are the platform error angles, $\delta v_{E},\delta v_{N},\delta v_{U}$ are the velocity errors in the ENU direction output by the INS of the follower UAV, δL,δλ,δh are the latitude, longitude and height error output by it, respectively, $\varepsilon_{bx},\varepsilon_{by},\varepsilon_{bz}$ are the constant drifts of the gyro of the INS and $\nabla_{bx},\nabla_{by},\nabla_{bz}$ is the bias of the accelerometer of the INS. Then, the state equation of the two sub-filters is:

(2) $$\dot{X}(t)=F(t)X(t)+G(t)W(t)$$

where F(t) is the state transition matrix, G(t) is the system noise driving matrix and W(t) is the system noise vector. 

### 3.2. Measurement Equation of Sub-Filters

#### 3.2.1. Measurement Equation of Sub-Filter 1 

Sub-filter 1 is the position loose integration filter of the INS/GPS. The position difference between the INS and the GPS is selected as the observation, and the position of the INS is expressed as:

(3) $$\left\{ \begin{array}{l} L_{INS}=L_{t}+\delta L\\ \lambda_{INS}=\lambda_{t}+\delta \lambda \\ h_{INS}=h_{t}+\delta h \end{array}\right.$$

The position of the GPS is expressed as:

(4) $$\left[\begin{array}{l} L_{GPS}\\ \lambda_{GPS}\\ h_{GPS} \end{array}\right]=\left[\begin{array}{l} L_{t}\\ \lambda_{t}\\ h_{t} \end{array}\right]-V_{GPS}$$

where $L_{t}$, $\lambda_{t}$ and $h_{t}$ represent the true values of latitude, longitude and height, and $V_{GPS}$ represents the measurement noise of the GPS in three directions of latitude, longitude and height. Then, the measurement equation of sub-filter 1 is:

(5) $$Z_{1}(t)=\left[\begin{array}{l} L_{INS}-L_{GPS}\\ \lambda_{INS}-\lambda_{GPS}\\ h_{INS}-h_{GPS} \end{array}\right]=H_{1}(t)X(t)+V_{1}(t)$$

where the measurement matrix $H_{1}(t)$ is:

(6) $$H_{1}(t)={\left[0_{3\times 6}\;I_{3\times 3}\;0_{3\times 6}\right]}_{3\times 15}$$

The noise vector $V_{1}(t)$ of the measurement system is equal to $V_{GPS}$, which is composed of the positioning error of the GPS in three directions. It is expressed as:

(7) $$V_{1}(t)={\left[\delta L_{GPS}\;\delta \lambda_{GPS}\;\delta h_{GPS}\right]}^{T}$$

#### 3.2.2. Measurement Equation of Sub-Filter 2

Sub-filter 2 is a filter that combines the output position a of the follower UAV’s INS, the range b measured by the UWB and the high-precision position c of the leader UAV. The measured value can be expressed as c−a−b. The range b is measured in the body coordinate system. If the UWB ranging module has been calibrated before installation and coincides with the body coordinate system of the follower UAV, the range b can be expressed as follows:

(8) $$P_{d}=\left[\begin{array}{l} P_{x}^{t}\\ p_{y}^{t}\\ P_{z}^{t} \end{array}\right]-V_{UWB}$$

where $P_{x}^{t}$, $P_{y}^{t}$ and $P_{z}^{t}$ represent the true values of the leader UAV in three directions under the body coordinate system of the follower UAV, and $V_{UWB}$ represents the random error of the UWB. Considering that the range between the follower UAV and the leader UAV is small, the Earth’s surface can be assumed to be a plane. And then, the relative range in the body coordinate system of the follower UAV can be expressed as follows:

(9) $$P_{L}-P_{W}^{INS}=C_{n}^{b}\left[\begin{array}{l} R\cos L_{INS}(\lambda_{L}-\lambda_{INS})\\ \;\;R(L_{L}-L_{INS})\\ \;\;h_{L}-h_{INS} \end{array}\right]$$

where R is the semi-major axis of the Earth, $L_{L}$, $\lambda_{L}$ and $h_{L}$ are the latitude, longitude and height position of the leader UAV obtained by the follower UAV, $L_{INS}$, $\lambda_{INS}$ and $h_{INS}$ are the latitude, longitude and height position output by the INS of the follower UAV and $C_{n}^{b}$ is the transition matrix from the ENU navigation coordinate system to the body coordinate system. Combining with Equation (3), Equation (9) can be further organized as:

(10) $$P_{L}-P_{W}^{INS}=\left[\begin{array}{l} P_{x}^{t}\\ p_{y}^{t}\\ P_{z}^{t} \end{array}\right]-C_{n}^{b}\left[\begin{array}{l} R\cos L_{INS}\delta \lambda \\ \;\;R\delta L\\ \;\;\;\;\delta h \end{array}\right]$$

Therefore, the measurement equation of sub-filter 2 is:

(11) $$Z_{2}(t)=P_{L}-P_{W}^{INS}-P_{d}=H_{2}(t)X(t)+V_{2}(t)$$

where the measurement matrix $H_{2}(t)$ is:

(12) $$H_{2}(t)={\left[0_{3\times 6}\;-C_{n}^{b}\left[\begin{array}{l} \;0\;R\cos L_{INS}\;0\\ R\;\;\;0\;\;\;0\\ \;0\;\;\;0\;\;\;1 \end{array}\right]\;0_{3\times 6}\right]}_{3\times 15}$$

The noise vector $V_{2}(t)$ is equal to $V_{UWB}$.

## 4. Design of the Adaptive Fault-Tolerant Federated Filter

**Figure 3:**
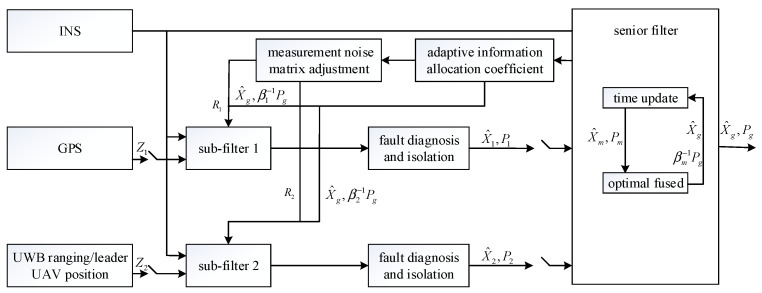
Structure of the adaptive fault-tolerant federated filter.

Based on the model established above, in order to reduce the influence of faults on global estimation and improve the robustness of the cooperative positioning system, an adaptive fault-tolerant federated filter, shown in Figure 3, is designed.

### 4.1. Fault Detection and Isolation

Aiming at a gradual fault in the GPS, the $\chi^{2}$ test method based on the residual is adopted to diagnose the gradual fault and isolate the faulty subsystems to improve the fault tolerance performance [25]. The residual of the sub-filter i at time k is:

(13) $$r_{i,k}=Z_{i,k}-H_{i,k}{\hat{X}}_{i,k/k-1}$$

When no fault occurs, the residual $r_{i,k}$ is a Gaussian white noise with a zero mean, and its variance is:

(14) $$A_{i,k}=H_{i,k}P_{i,k/k-1}H_{i,k}^{T}+R_{i,k}$$

where the fault detection function is:

(15) $$\lambda_{i,k}=r_{i,k}^{T}A_{i,k}^{-1}r_{i,k}$$

where $\lambda_{i,k}$ is the distribution of $\chi^{2}$ with three degrees of freedom. The fault decision criterion is:

(16) $$\left\{ \begin{array}{l} \lambda_{i,k}>T_{D}\;\;\;fault\\ \lambda_{i,k}\leq T_{D}\;\;\;no\;fault \end{array}\right.$$

where $T_{D}$ is the preset threshold, which is determined by the false alarm probability $P_{f}$. If sub-filter 1 fails, it does not share in the global fusion. If sub-filter 2 fails, another leader UAV needs to be selected for ranging.

### 4.2. Adaptive Measurement Noise Matrix Adjustment

The $\chi^{2}$ test method based on the residual has a detection delay for gradual faults, which may result in missed detections [26]. At this time, the faulty sub-filter will pollute the global estimation. In order to reduce the influence of the faulty sub-filter on the global estimation before isolating the faulty sub-filter, the measurement noise matrix of the sub-filter is adjusted by the adaptive information allocation coefficient to change the degree of utilization of the observed information. The information allocation coefficient depends on the observation forecast residual [27], and the sub-filter information allocation coefficient is:

(17) $$\beta_{i,k}=\left\{ \begin{array}{l} 1\;\;\;\;\;\;\left|\Delta V_{i,k}\right|\leq d\\ \frac{d}{\left|\Delta V_{i,k}\right|}\;\;\;\left|\Delta V_{i,k}\right|>d\; \end{array}\right.\;$$

where d is generally taken as 0.85~0.1, and 0.85 is taken in this paper. $\Delta V_{i,k}$ is a statistic constructed by the residual $r_{i,k}$. $r_{i,k}$ can be expressed as:

(18) $$r_{i,k}=Z_{i,k}-H_{i,k}{\hat{X}}_{i,k/k-1}$$

Then, the expression of $\Delta V_{i,k}$ is:

(19) $$\Delta V_{i,k}=\frac{r_{i,k}^{T}r_{i,k}}{tr(H_{i,k}P_{i,k/k-1}H_{i,k}^{T}+R_{i,k})}$$

Since the information allocation coefficient satisfies the principle of information conservation, it is necessary to normalize the allocation coefficient obtained in Equation (17):

(20) $$\beta_{i,k}^{t}=\frac{\beta_{i,k}}{\sum_{i=1}^{2}\beta_{i,k}}$$

where $\beta_{i,k}^{t}$ is the information allocation coefficient of the normalized sub-filter i at time k. When the observation information of the sub-filter is fault-free, the information allocation coefficient is 0.5, and the measurement noise matrix is the same as the initial measurement noise matrix. When the observation information of the sub-filter is faulty, the utilization of the observation information should be reduced by increasing the measurement noise matrix. On the contrary, the fault-free sub-filter needs to increase the utilization of its own observation information by reducing the measurement noise matrix, thereby reducing the impact of the feedback global estimation on its own accuracy. The adjustment of the measurement noise matrix can be determined by the number of sub-filters and the information allocation coefficient, so the measurement noise matrix of sub-filter i at time k is:

(21) $$R_{i,k}=R_{i}+2(0.5-\beta_{i,k}^{t})R_{i}$$

where $R_{i}$ is the initial measurement noise matrix, and 2 represents the number of sub-filters.

In the adaptive fault-tolerant federated filter designed in this paper, sub-filter 1 based on the INS/GPS and sub-filter 2 based on the INS/UWB ranging position of the leader UAV run in parallel. Because the state quantities of the two sub-filters are the same, the time update of each sub-filter is performed in the main filter, and the measurement update is still performed in each sub-filter. In addition, the independence and irrelevance of each sub-filter’s state estimation can be guaranteed without changing it by the variance upper bound technique and the information allocation principle. And then, the state estimation of the sub-filter and the covariance matrix are fused to achieve global optimal estimation. The specific design steps of the adaptive fault-tolerant federated filter are as follows:

(1) Information allocation. Since the master filter in the adaptive fault-tolerant federated filter does not allocate information, according to the principle of information conservation, the information allocation coefficient is:

(22) $$\beta_{m}=0,\sum_{i=1}^{2}\beta_{i}^{t}=1$$

where $\beta_{m}$ is the information allocation coefficient of the master filter, and $\beta_{i}^{t}$ is the information allocation coefficient of the sub-filter i. The calculation formula for $\beta_{i}^{t}$ is shown in Equation (20). The global state estimation, covariance matrix and process noise matrix are assigned to each sub-filter, that is:

(23) $$\left\{ \begin{array}{l} {\hat{X}}_{i,k-1}={\hat{X}}_{g,k-1}\\ P_{i,k-1}={\left(\beta_{i}^{t}\right)}^{-1}P_{g,k-1}\\ Q_{i,k-1}={\left(\beta_{i}^{t}\right)}^{-1}Q_{f} \end{array}\right.$$

where ${\hat{X}}_{i,k-1}$ is the state estimate of the sub-filter i at time k−1, $P_{i,k-1}$ is the covariance matrix of the sub-filter i at time k−1, $Q_{i,k-1}$ is the process noise matrix of the sub-filter i at time k−1 and ${\hat{X}}_{g,k-1}$, $P_{g,k-1}$ and $Q_{f}$ are the global state estimate, covariance matrix and process noise matrix.

(2) Time update of each sub-filter in the master filter. The state equation and measurement equation of each sub-filter are discretized before the time update as follows:

(24) $$X_{i,k}=\Phi_{i,k,k-1}X_{i,k-1}+\Gamma_{i,k-1}W_{i,k-1}$$

(25) $$Z_{i,k}=H_{i,k}X_{i,k}+V_{i,k}$$

where $\Phi_{i,k,k-1}$ is the discrete state transition matrix, $X_{i,k}$ is the state estimate of the sub-filter i at time k, $Z_{i,k}$ is the measurement of the sub-filter i at time k, $H_{i,k}$ is the measurement matrix of the sub-filter i at time k,$\Gamma_{i,k-1}$ is the noise driving matrix of the sub-filter i at time k−1 and $W_{i,k-1}$ and $V_{i,k}$ are the system noise and measurement noise of the sub filter i at time k−1 and k, respectively. Then, the time update of each sub-filter in the master filter can be expressed as follows:

(26) $${\hat{X}}_{i,k/k-1}=\Phi_{i,k,k-1}{\hat{X}}_{i,k-1}$$

(27) $$P_{i,k/k-1}=\Phi_{i,k,k-1}P_{i,k-1}\Phi_{i,k,k-1}^{T}+\Gamma_{i,k-1}Q_{i,k-1}\Gamma_{i,k-1}^{T}$$

where ${\hat{X}}_{i,k/k-1}$ is the one-step prediction of the state of the sub-filter i from time k−1 to time k. $P_{i,k/k-1}$ is the one-step prediction of the covariance matrix of the sub-filter i from time k−1 to time k.

(3) Independent measurement updates for each sub-filter. Each sub-filter processes its own measurement information, and the measurement updates as follows:

(28) $$K_{i,k}=P_{i,k/k-1}H_{i,k}^{T}{\left(H_{i,k}P_{i,k/k-1}H_{i,k}^{T}+R_{i,k}\right)}^{-1}$$

(29) $${\hat{X}}_{i,k}={\hat{X}}_{i,k/k-1}+K_{i,k}(Z_{i,k}-H_{i,k}{\hat{X}}_{i,k/k-1})$$

(30) $$P_{i,k}=(I-K_{i,k}H_{i,k})P_{i,k/k-1}$$

where $R_{i,k}$ is the measurement noise matrix of the sub-filter i at time k. $R_{i,k}$ needs to be adaptively adjusted before the measurement is updated, and its calculation formula is shown in Equation (21).

(4) The global optimal estimation of the master filter fused with the local estimation of the sub-filter. Before fusion, each sub-filter needs to perform fault detection and isolation steps, and the fault sub-filter does not participate in the fusion. The master filter obtains global optimal state estimates based on the estimates of each sub-filter, which is optimally synthesized according to Equation (31):

(31) $$\left\{ \begin{array}{l} P_{g,k}={\left(\sum_{i=1}^{2}P_{i,k}^{-1}\right)}^{-1}\\ {\hat{X}}_{g,k}=P_{g,k}(\sum_{i=1}^{2}P_{i,k}^{-1}{\hat{X}}_{i,k}) \end{array}\right.$$

where $P_{g,k}$ is the covariance matrix of the global estimation error at time k, and ${\hat{X}}_{g,k}$ is the optimal estimates of the global error states at time k. The global estimation at time k has been completed and returned to step 1 the next time.

## 5. Simulation Verification and Analysis

### 5.1. Simulation Conditions Set

In the multi-UAV formation, all the leader UAVs are equipped with the same high-precision INS/GPS integrated navigation equipment and UWB ranging module, and all the follower UAVs are equipped with the same low-precision GPS, low-precision INS navigation equipment and UWB ranging module. In addition, there is a wireless communication network between the leader UAVs and the follower UAVs, which ensures that the follower UAVs can obtain the high-precision position of the leader UAV. The simulation conditions are set as shown in Table 1. The settings of the simulation conditions are consistent with the typical values in real situations. After setting the simulation conditions, they are imported into the PINS navigation toolbox to generate the data needed for simulation. The PINS navigation toolbox can ensure the reliability of data sources.

### 5.2. Simulation Results and Analysis

**Figure 4:**
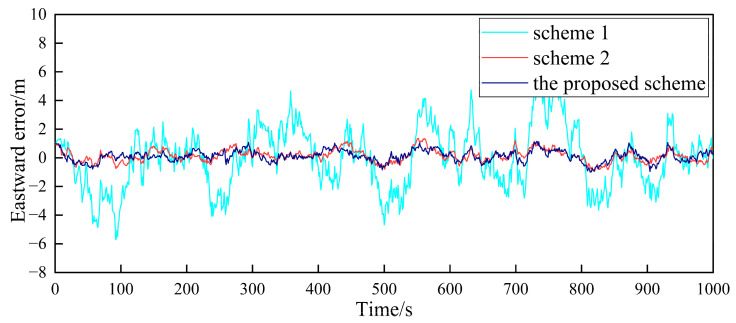
Comparison of the eastward positioning error of follower UAV 1.

**Figure 5:**
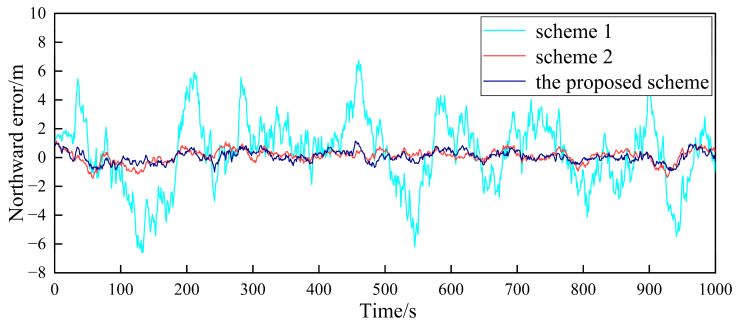
Comparison of the northward positioning error of follower UAV 1.

**Figure 6:**
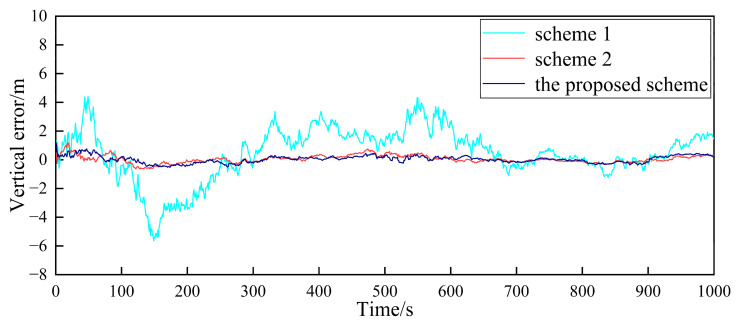
Comparison of the vertical positioning error of follower UAV 1.

**Figure 7:**
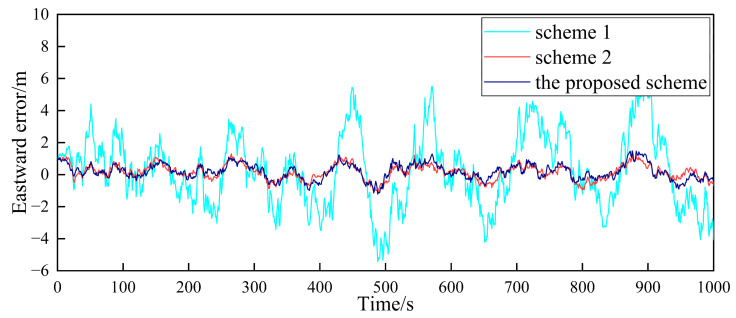
Comparison of the eastward positioning error of follower UAV 2.

**Figure 8:**
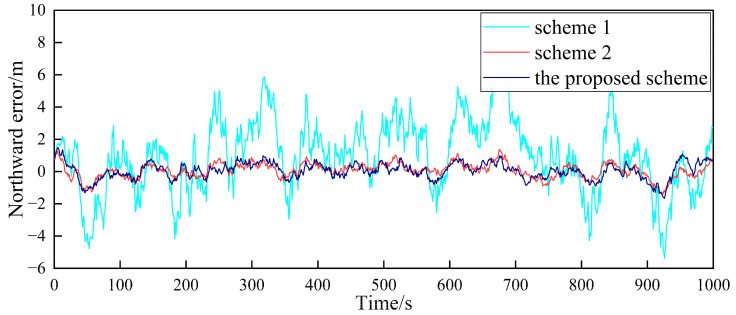
Comparison of the northward positioning error of follower UAV 2.

**Figure 9:**
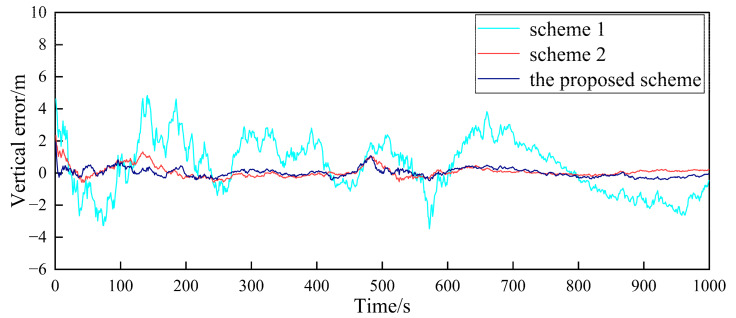
Comparison of the vertical positioning error of follower UAV 2.

Based on the above simulation conditions, two follower UAVs and the nearest leader UAV in the multi-UAV formation are selected to simulate the cooperative positioning of the follower UAV. The filter frequency of the sub-filter and the master filter is set as 1Hz. MATLAB was used to verify the feasibility of the algorithm. First, in the case where the GPS of the follower UAV does not fail, Figure 4, Figure 5, Figure 6, Figure 7, Figure 8 and Figure 9 show the comparison of the positioning error of follower UAV 1 and follower UAV 2 in three directions by three different schemes, respectively:

(1) Scheme 1: The follower UAV only relies on its own low-precision GPS and INS for positioning, without range measurement or information interaction with the leader UAV.

(2) Scheme 2: The follower UAV adopts the traditional federated filter with fault detection and isolation for cooperative positioning. The information allocation coefficients of the two sub-filters satisfy the principle of equal division. The measurement noise matrix does not change.

(3) The proposed scheme: The follower UAV adopts the adaptive fault-tolerant federated filter designed in this paper for collaborative positioning. Different from Scheme 2, this scheme can adjust the measurement matrix of the sub-filter through the adaptive information allocation coefficient so as to determine the degree of influence of the observed information on the estimated results.

It can be seen from Figure 4, Figure 5, Figure 6, Figure 7, Figure 8 and Figure 9 that the positioning errors in three directions are relatively large when only the low-precision navigation equipment of the follower UAV is adopted for positioning without GPS failure. The positioning accuracy is greatly improved when Scheme 2 and the proposed scheme in this paper for cooperative positioning are adopted. In order to further analyze the influence of the three schemes on the positioning accuracy of the follower UAV, the root mean square error (RMSE) and mean based on the three schemes of the follower UAV 1 are counted, as shown in Table 2.

According to Figure 4, Figure 5, Figure 6, Figure 7, Figure 8 and Figure 9, combined with the results shown in Table 2, the results show that, under the condition of normal observation, the positioning errors of the follower UAV are optimally estimated by Scheme 2 and the proposed scheme in this paper for cooperative positioning, which can greatly improve the positioning accuracy of the follower UAV, and it is close to that of the leader UAV. Shown as RMSE, the positioning accuracy is improved by 81.7% when Scheme 2 is adopted for cooperative positioning, and the positioning accuracy is improved by 83.4% when the proposed scheme in this paper is adopted. Considering the RMSE and mean, the positioning accuracies of Scheme 2 and the proposed scheme in this paper are basically the same when no fault occurs.

**Figure 10:**
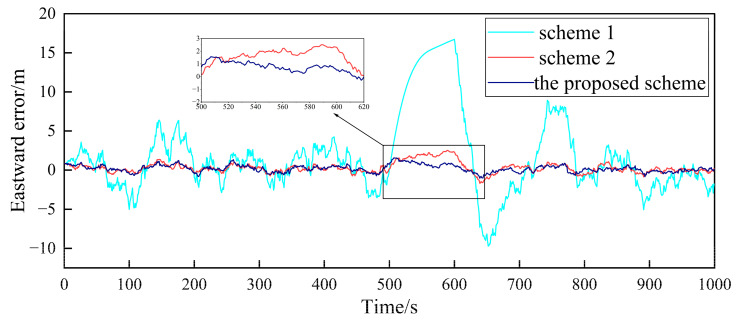
Comparison of the eastward positioning error of follower UAV 1.

**Figure 11:**
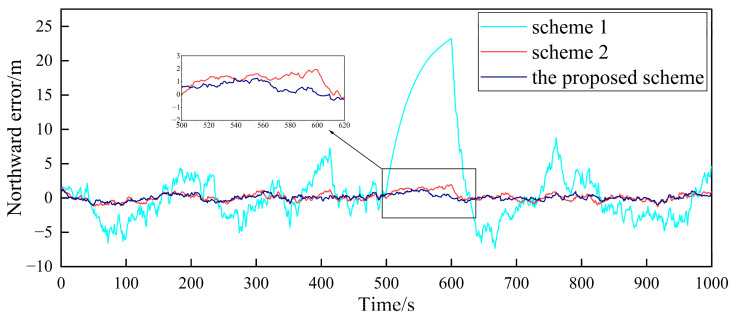
Comparison of the northward positioning error of follower UAV 1.

**Figure 12:**
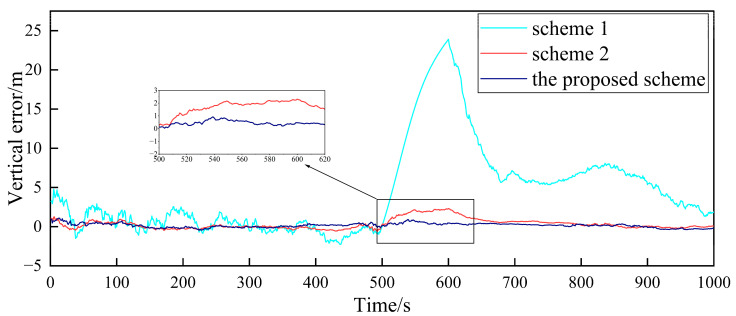
Comparison of the vertical positioning error of follower UAV 1.

In order to verify the fault-tolerance performance of the three schemes, it is simulated for the case of the gradual GPS failure of the follower UAV. It is assumed that after flying for 500 s, the GPS of follower UAV 1 has a gradual fault. The fault rate in the three directions is 0.1 m/s, and the duration is 100 s. The false alarm probability is 0.01, so the detection threshold is $T_{D}=11.34$. The simulation results are shown in Figure 10, Figure 11 and Figure 12.

Figure 10, Figure 11 and Figure 12, respectively, show the comparison of the positioning errors of two different schemes when the gradual fault occurs. It is shown that when the gradual fault occurs, the positioning accuracy and fault tolerance of Scheme 1 are very poor in the three directions, while Scheme 2 and the proposed scheme in this paper have a good fault tolerance, but the error of the proposed scheme in the data abnormal time period of 500–600 s is lower than that of Scheme 2. In order to quantitatively analyze the fault tolerance performance of Scheme 2 and the proposed scheme in this paper, the RMSE and mean are counted, as shown in Table 3.

The results in Table 3 show that the RMSE and mean in the three directions of the proposed scheme in this paper are lower than those of Scheme 2 during the 500–600 s when the fault occurs. This shows that the positioning accuracy of the proposed scheme for cooperative positioning is significantly higher than that of Scheme 2 after the occurrence of the fault. The proposed scheme has a stronger fault tolerance than Scheme 2, which is mainly due to the adaptive adjustment of the measurement noise matrix. When the fault occurs, the proposed scheme can effectively reduce the influence of the fault on global estimation by adjusting the measurement noise matrix. Finally, the comparison of the elapsed time of each scheme is shown in Table 4 to compare the accuracy improvement cost of multi-UAV positioning. 

From Table 4, it can be seen that the consumption times of Scheme 2 and the proposed scheme are longer than that of Scheme 1, and the consumption times of Scheme 2 and the proposed scheme are basically the same. In general, the difference in the calculation amount of the three schemes is not very large and is within the acceptable range.

Based on the above simulation results, it can be seen that the proposed scheme in this paper balances the positioning accuracy and fault-tolerance performance of the federated filter by adjusting the measurement noise matrix, and the consumption time of the proposed scheme is basically consistent with that of Scheme 2. The proposed scheme in this paper can meet the requirements of the cooperative positioning accuracy of the follower UAV, and it can also make the cooperative positioning process of the follower UAV have a strong fault tolerance performance. 

## 6. Conclusions

Aiming at the cooperative positioning problem of multi-UAV formation, a method based on an adaptive fault-tolerant federated filter is studied. This method adopts the $\chi^{2}$ test method based on the residual to detect and isolate the gradual faults. Considering that the $\chi^{2}$ test method based on the residual has a detection delay for the gradual fault, the adaptive information allocation coefficient is used to adjust the measurement noise matrix to reduce the impact of the fault on the global estimation in this paper. The simulation results show that the positioning accuracy of the adaptive fault-tolerant federated filter algorithm is greatly improved compared to the absolute positioning accuracy of a single UAV, which is close to the positioning accuracy of the leader UAV. In addition, compared with the traditional fault-tolerant federated filter, the adaptive fault-tolerant federated filter designed in this paper can better reduce the impact of gradual faults on the global estimation, which is suitable for the flight positioning scene of a multi-UAV formation in complex environments.

## Figures and Tables

**Table 1:** Simulation conditions.

UAVs	Items	Data
Leader UAVs	INS/GPS eastward error	1 m
	INS/GPS northward error	1 m
	INS/GPS vertical error	1 m
	UWB ranging noise	0.03 m
Follower UAVs	GPS eastward error	10 m
	GPS northward error	10 m
	GPS vertical error	10 m
	Gyro drift	1 deg/h
	Gyroscope random walk	0.1${}^{\circ}/\sqrt{h}$
	Accelerometer bias	100 μg
	Accelerometer random walk	5 $\mu g/\sqrt{\mathrm{Hz}}$
	UWB ranging noise	0.03 m

**Table 2:** RMSE and mean of positioning errors of follower UAV 1.

Positioning Error	m			m		
	Scheme 1	Scheme 2	The Proposed Scheme	Scheme 1	Scheme 2	The Proposed Scheme
Eastward	2.10	0.45	0.41	1.68	0.35	0.33
Northward	2.43	0.45	0.40	1.99	0.36	0.32
Vertical	1.85	0.27	0.25	1.44	0.20	0.20

**Table 3:** RMSE and mean comparison among 500–600 s.

Positioning Error	m		m	
	Scheme 2	The Proposed Scheme	Scheme 2	The Proposed Scheme
Eastward	1.84	0.95	0.54	0.40
Northward	1.34	0.76	0.49	0.36
Vertical	1.77	0.50	0.49	0.26

**Table 4:** Comparison of the elapsed time of each scheme.

Simulation	Elapsed Time/s		
	Scheme 1	Scheme 2	The Proposed Scheme
No fault	0.23	0.94	0.95
Fault	0.25	0.96	0.98

## Data Availability

Data are available from the PSINS open-source toolbox.
